# Trench Foot: Its Treatment

**Published:** 1918-12

**Authors:** Baily K. Ashford


**Trench Foot : Its Treatment**. By Colonel Bailey K. Ashford, M. C., formerly Commandant, Army Sanitary School.

The speaker said in part :

My own personal experience with trench foot was brief but illuminating, as showing :

(1) The power for disabling the soldier inherent in this affection.

(2) The rapidity with which Raymond's treatment of early cases rendered men so affected fit for duty.

(3) The value of interallied commerce of ideas by means of our schools.

In September, 1917, I made a trip to the French Sanitary School preparatory to assuming the duties of Commandant of a Corps training school for our own medical officers. I was there only a few hours, but just before leaving, Colonel Victor Raymond, of the Service de Santé of the French Army, gave me a brief resumé of trench foot and his brochure, which was the first notion I had had of the disease.

A training school for line officers was established in the damp October chill we all remember so well last year. Our division had arrived in July at their training area, and as most of us had been serving in tropical or subtropical countries we had little idea of the climate of the Haute-Marne. The army was just organizing, and we had little transportation and comparatively little wood for fires. A barrack cantonment for the line school sprang up overnight in a beautiful meadow, which in forty-eight hours became a sea of mud. The temperature ran between zero and 10° C. There had been no time to construct drying rooms. It rained for forty days and forty nights with persistence, and men and officers were so intent upon learning, that they forgot mud, water, and cold and trudged about all day with wet feet without sufficient means for drying their shoes at night. The result was that every one in a few days had all their foot-wear muddy and soaked through, and were obliged to put on wet shoes in the morning. A battalion on duty at this camp, a half mile from the town, were engaged in construction and were constantly wet through. From the very first, the commanding officer had been going to great lengths to secure fuel and to prevent the effects of exposure; but before anyone had time to do anything, the camp surgeon, on the first day I was given medical supervision over this camp, called my attention to a prevalent affection of the feet of the men working there, as well as of the student officers. These men had not been in the trenches, but the surgeon stated that the clinical picture resembled what he had seen described somewhere as "trench foot", and called me in to go over the situation. By that time the battalion was seriously crippled by a large number of men who were actually unable to walk with any degree of comfort. I went over this organization, man by man, and found that a good twenty-five per cent. had what they called "chilblains". One or two men had gone to hospital with beginning necrosis, and those in quarters at the camp, as well as many still trying to do duty, were in the first or second stage of the affection.

That same day Colonel Raymond's brochure was abstracted, particularly that part relating to prophylaxis and treatment; efforts were redoubled, and this time with success, to provide more fuel; and the condition was reported to the Chief Surgeon with the diagnosis of trench foot, one of the first, if not the very first, made in our Army. Telegraphic authority was requested, and promptly obtained, for a good supply of whale oil, camphor, borate of soda, and green soap, secured in open market, and within three or four days the outbreak had declined as suddenly as it had appeared.

I have detailed the conditions under which the affection appeared, because the sudden and severe onset was evidently precipitated by those conditions, and troops who have to live under them here do not have to be in trenches to reap the results of exposure to moderate cold, with constantly wet and muddy foot-wear. The disease made its appearance under the very conditions considered by Raymond as favoring its development. It was a short lesson, but a striking one, at a propitious time for the American Army, and thanks to the energetic action of the Chief Surgeon of the American E. F. in acquainting the entire Army with its prevention and cure, it has never shown its head since as an important source of invaliding of soldiers.

In the meantime, we had personally followed the cases, and had carried into effect the prophylaxis. In all about 300 cases of some 1000 strength were treated, all of which were promptly and completely cured without loss of tissue. In fact, not over three men had beginning necrosis, 15 o/o blisters, and the rest merely the edematous, or first stage. The most notable result was seen in the rapid return to normal of all treated by the camphor and sodium borate fomentations. In forty-eight hours they were usually free from symptoms and ready for duty.

*Preventive Treatment.*

The first circular on this subject made at the time, and embodying all of the essential features of the disease, was published by the Army Sanitary School, and is still issued. Herein was recommended.

(1) The daily examination of the feet in each squad by squad leaders. All men with " sore, red, and swollen feet " were to be sent promptly to the surgeon for inspection.

(2) The daily massage of the feet with camphorated oil furnished by the Surgeon. This was to be done in the presence of the squad leader, preferably before, or just after, breakfast.

(3) On relief from duty each day men were to be made to wash their feet with a soap composed of borate of soda 10, camphor 2.5, and green soap 100, dry them and powder them with our army foot powder (salicylic acid, starch and talcum) plus 2.5 o/o of camphor. After this, dry socks were to be donned.

(4) Provision for drying shoes, and clothing.

(5) Furnishing of well-fitting shoes, presence of officers during issue of same, and greasing of all shoes and boots.

The division nearby, having just received its order to go to the trenches for the first time, were enjoined to carry out the provisions of the above circular, when in rest and when actually in the trenches for the first time, and were enjoined to carry the following recommendations into effect.

(1) Good drainage of trenches and careful duckboard flooring of same.

(2) Provision of three pairs of greased socks per man.

(3) Massage of feet twice a day with camphorated oil or camphorated whale oil. Exercise of feet and toes from time to time, and always as often as possible if numbness should be perceived.

(4) Avoidance of warming affected feet before a fire.

(5) Prohibition of rubber boots except when men actually had to stand in muddy water for an extended period.

These measures saved the division from the fate of the training school, as practically no cases were reported, although much worse predisposing conditions prevailed. They also cut short the outbreak in the school itself. Certainly some of these recommendations would today be simplified, as the manifest impossibility of securing compliance with so elaborate a plan is recognized, but it must be remembered that we were under the influence of the dramatic entry of a new and disabling affection at a serious time, and we " went the limit. "

This was followed by a circular from our G. H. Q., which in addition emphasized :

(1) That trench foot is preventable, and that organization commanders are held directly responsible for its prevalence in their commands.

(2) That insufficient and cold food, plus lack of sleep and comfort, as well as lack of cleanliness of the feet, predispose to this disease.

(3) That constriction by leggings, puttees, and tight shoes was a fruitful cause of trench foot.

Commanding officers were also enjoined, in addition to what was recommended in our first circular, as follows :

(1) That dry woolen socks should be furnished their men.

(2) That garters be forbidden, the socks being pinned up with safety pins.

(3) That at least one change of shoes or boots be carried by each soldier and officer.

(4) That rubber boots be not worn, save for a period of a few hours, and then only when obliged to stand in muddy water.

(5) That suitable drying chambers be established.

(6) That dry woollen socks be delivered to the men in the trenches daily with return of wet ones to the drying room.

Since that time of historical interest, whale oil and grease in general has begun to be looked upon with disfavor. The odor is bad, secretions are held back, the feet are exposed to maceration and then locked up. Saprophytic organisms accommodate themselves to their new surroundings and invade the tissues, increasing rather than preventing this affection.

Whatever may be the predisposing cause of trench foot, and even though only in the presence of such predisposing conditions does trench foot arise, it should not be forgotten that invasion of devitalized tissue by saprophytes and subsequent serious infection is at least a serious and frequent complication, and an ever-present menace locally — at times generally — in trench foot. Hence cleanliness of the feet, vitalizing measures, and drugs deterrent to the development of certain saprophytes locally applied are of tremendous importance.

### *The Treatment of Cases.*

*Ist Stage — Edematous Stage.*

Foot baths with green soap and water.

Large Fomentations (hot) of :

| | |
|---|---|
| Camphor | 1. |
| Borate of soda | 15. |
| Boiled water | 1000. |

The gauze compresses should be covered with an impermeable material (oiled silk or oiled paper) and should extend well above the upper limit of the edema. They are to be continued until the edema notably subsides. The most striking early effect of this treatment is the relief from pain. Men who have been unable to rest on this account usually drop off to sleep quite promptly.

The use of potassium iodide has an almost specific action in relieving pain in the feet and legs due to trench foot.

*2nd Stage — Blisters.*

Cut away the raised epithelium of all blebs larger than a fifty centime piece, and wipe away gently the gelatinous floor. Then cover with a compress of :

| | |
|---|---|
| Camphor | 30. |
| Ether | 1000. |

Over this compress place the fomentations, as per treatment of the first stage.

*3rd Stage — Sloughs.*

Avoid operative surgery. Loosen sloughs by dressings, as per treatment of the second stage, or use the paraffin foot-bath. This is most excellent treatment, but must be applied in a well appointed hospital to which such cases are apt to be sent. It consists simply in the immersion, for from one-half to one hour, of the feet in a small foot bath filled with paraffin at 60° C., or higher. After this the cotton batting, in which the feet are wrapped before immersion, is left on; the paraffin it takes up is solidified, and the result is a veritable paraffin sabot, which is left on. This should be done at first daily, afterward every other day. I saw this treatment carried out at the Saint Nicolas Hospital in Paris with excellent results.

When sloughs are hard, incision should be made through them down to the fetid gelatinous layer below, and the compresses then applied, but blood should not be drawn, nor, especially, the cautery used.

When granulations need stimulating Raymond recommends :

| | |
|---|---|
| Eucalyptol / Balsam peruv. / Guaiacol (each) | 10. |
| Camphor | 1000. |

He states that this can be used by painting locally, and alternating this with the usual fomentations above described, or paraffin foot-baths.

Above all, do not forget to feed up your patients. They are very apt to be weak, mal-nourished, exhausted individuals.

Finally, tetanus antitoxin should *always* be administered to all patients beyond the first stage, and repeated every 8 days until the lesion looks healthy. Trench foot predisposes strongly to tetanus, and neglect of this warning will certainly cause some preventable deaths.

